# 3-Amino-5,5-di­phenyl­imidazolidine-2,4-dione

**DOI:** 10.1107/S1600536814002487

**Published:** 2014-02-08

**Authors:** Joel T. Mague, Alaa A.-M. Abdel-Aziz, Adel S. El-Azab

**Affiliations:** a Department of Chemistry, Tulane University, New Orleans, LA 70118, USA; b Department of Pharmaceutical Chemistry, College of Pharmacy, King Saud University, Riyadh 11451, Saudi Arabia; c Department of Medicinal Chemistry, Faculty of Pharmacy, University of Mansoura, Mansoura 35516, Egypt; d Department of Organic Chemistry, Faculty of Pharmacy, Al-Azhar University, Cairo 11884, Egypt

## Abstract

The title compound, C_15_H_13_N_3_O_2_, crystallizes with two independent mol­ecules in the asymmetric unit, which differ considerably in the dihedral angles made between the phenyl groups and the five-membered rings [47.19 (8) and 61.16 (9)° in one mol­ecule and 55.04 (10) and 55.00 (8)° in the other]. In the crystal, N—H⋯O hydrogen bonds generate columnar units having approximate fourfold rotational symmetry about axes parallel to *b*.

## Related literature   

For the biological properties of hydantoins, see: El-Deeb *et al.* (2010 ▶); Rajic *et al.* (2006 ▶); Carmi *et al.* (2006 ▶); Sergent *et al.* (2008 ▶). For the preparation of the title compound, see: Kiec-Kononowicz *et al.* (1984 ▶). For related crystal structures, see: Delgado *et al.* (2007 ▶); Roszak & Weaver (1998 ▶); Kashif *et al.* (2008 ▶); Coquerel *et al.* (1993 ▶); SethuSankar *et al.* (2002 ▶); Eknoian *et al.* (1999 ▶); Ciechanowicz-Rutkowska *et al.* (1994 ▶).
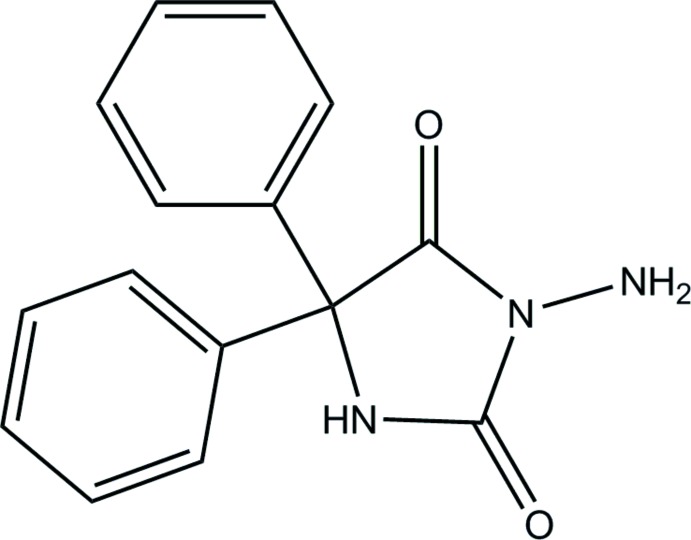


## Experimental   

### 

#### Crystal data  

C_15_H_13_N_3_O_2_
*M* *_r_* = 267.28
Monoclinic, 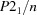

*a* = 20.1565 (7) Å
*b* = 6.1651 (2) Å
*c* = 20.3250 (7) Å
β = 97.781 (2)°
*V* = 2502.47 (15) Å^3^
*Z* = 8
Cu *K*α radiation
μ = 0.79 mm^−1^
*T* = 100 K
0.22 × 0.07 × 0.05 mm

#### Data collection  

Bruker D8 VENTURE PHOTON 100 CMOS diffractometer
Absorption correction: multi-scan (*SADABS*; Bruker, 2012 ▶) *T*_min_ = 0.84, *T*_max_ = 0.96
32772 measured reflections
4560 independent reflections
3185 reflections with *I* > 2σ(*I*)
*R*_int_ = 0.105

#### Refinement  

*R*[*F* ^2^ > 2σ(*F* ^2^)] = 0.054
*wR*(*F* ^2^) = 0.111
*S* = 1.08
4560 reflections
361 parameters
H-atom parameters constrained
Δρ_max_ = 0.22 e Å^−3^
Δρ_min_ = −0.24 e Å^−3^

### 

Data collection: *APEX2* (Bruker, 2012 ▶); cell refinement: *SAINT* (Bruker, 2012 ▶); data reduction: *SAINT*; program(s) used to solve structure: *SHELXS97* (Sheldrick, 2008 ▶); program(s) used to refine structure: *SHELXL97* (Sheldrick, 2008 ▶); molecular graphics: *DIAMOND* (Brandenburg & Putz, 2012 ▶); software used to prepare material for publication: *SHELXTL* (Sheldrick, 2008 ▶).

## Supplementary Material

Crystal structure: contains datablock(s) I, global. DOI: 10.1107/S1600536814002487/sj5389sup1.cif

Structure factors: contains datablock(s) I. DOI: 10.1107/S1600536814002487/sj5389Isup2.hkl

Supporting information file. DOI: 10.1107/S1600536814002487/sj5389Isup3.cml

CCDC reference:

Additional supporting information:  crystallographic information; 3D view; checkCIF report

## Figures and Tables

**Table 1:** Hydrogen-bond geometry (Å, °)

*D*—H⋯*A*	*D*—H	H⋯*A*	*D*⋯*A*	*D*—H⋯*A*
N1—H1⋯O1^i^	0.91	1.92	2.828 (3)	177
N3—H3*A*⋯O3	0.91	2.11	2.957 (3)	154
N4—H4⋯O4^ii^	0.91	1.91	2.820 (3)	176
N6—H6*A*⋯O3^i^	0.91	2.58	3.320 (3)	139
N6—H6*B*⋯O2^iii^	0.91	2.46	3.070 (3)	124
N6—H6*B*⋯N3^iii^	0.91	2.52	3.363 (3)	154

Symmetry codes: (i) 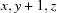; (ii) 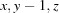; (iii) 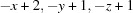.

**Fig. 1.**
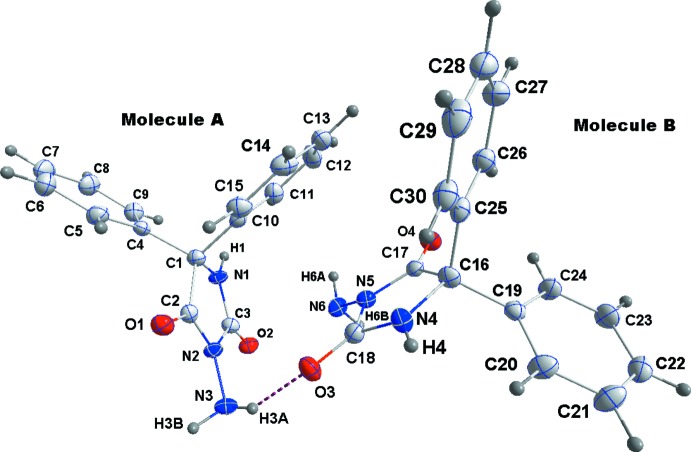
Perspective view of the asymmetric unit showing the N—H···O hydrogen bond between molecules A and B as a dashed line. Displacement ellipsoids are drawn at the 50% probability level.

**Fig. 2.**
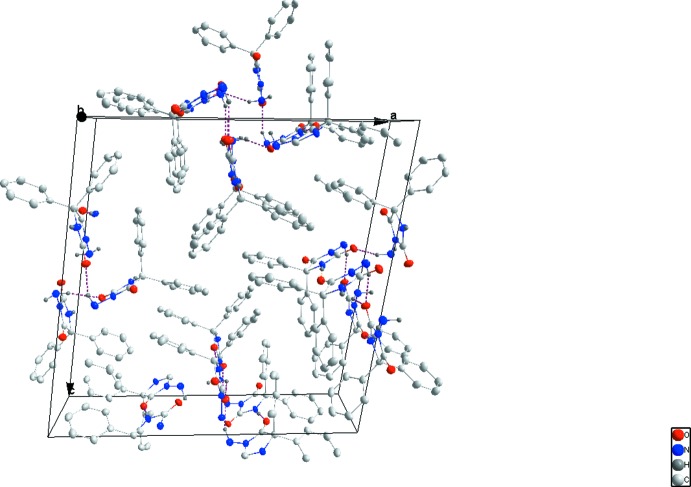
Packing diagram viewed perpendicular to (-1,6,-4) along with intermolecular N—H···O interactions shown as dotted lines.

## supplementary crystallographic information

### 1. Comment

Hydantoins are an important class of compounds which have long attracted attention, owing to their remarkable biological and pharmacological properties. These include antitumor and antiviral activity, insulinotropic properties and use as EGFR inhibitors. (El-Deeb *et al.*, 2010; Rajic *et al.*, 2006; Carmi *et al.*, 2006; Sergent *et al.*, 2008). Of several structure determinations of hydantoins (Delgado *et al.*, 2007; Kashif *et al.*, 2008; Coquerel *et al.*, 1993; SethuSankar *et al.*, 2002; Ciechanowicz-Rutkowska *et al.*, 1994; Roszak & Weaver, 1998; Eknoian *et al.*, 1999), those in the last two reports bear the greatest similarity to the title compound. The title compound crystallizes with two independent molecules (A and B) in the asymmetric unit. The 5-membered ring in A is planar to within 0.032 Å while that in B is planar to within 0.016 Å. Molecules A and B differ most notably in the dihedral angles which the pendant phenyl rings make with the 5-membered ring. For molecule A the angles for C4/C5/C6/C7/C8/C9 and for C10/C11/C12/C13/C14/C15 are, respectively, 47.19 (8) and 61.16 (9)°. In molecule B the angles for C19/C20/C21/C22/C23/C24 and for C25/C26/C27/C28/C29/C30 are identical at 55.04 (10) and 55.00 (8)°, respectively. Both the imino and amino groups participate in intermolecular N—H···O hydrogen bonding which forms columns having approximate 4-fold rotational symmetry about axes parallel to *b* (Table 1 and Fig. 2).

### 2. Experimental

The title compound, 3-amino-5,5-diphenylimidazolidine-2,4-dione, was successfully obtained by the reaction of 5,5-diphenylhydantoin and hydrazine hydrate following the published route (Kiec-Kononowicz *et al.*, 1984).

### 3. Refinement

H-atoms attached to carbon were placed in calculated positions (C—H = 0.95 Å) while those attached to nitrogen were placed in locations derived from a difference map and their coordinates adjusted to give N—H = 0.91 Å. All were included as riding contributions with isotropic displacement parameters 1.2 times those of the attached atoms.

### Crystal data

**Table:**

C_15_H_13_N_3_O_2_	*F*(000) = 1120
*M**_r_* = 267.28	*D*_x_ = 1.419 Mg m^−^^3^
Monoclinic, *P*2_1_/*n*	Cu *K*α radiation, λ = 1.54178 Å
*a* = 20.1565 (7) Å	Cell parameters from 9869 reflections
*b* = 6.1651 (2) Å	θ = 2.9–68.2°
*c* = 20.3250 (7) Å	µ = 0.79 mm^−^^1^
β = 97.781 (2)°	*T* = 100 K
*V* = 2502.47 (15) Å^3^	Needle, clear colourless
*Z* = 8	0.22 × 0.07 × 0.05 mm

### Data collection

**Table:**

Bruker D8 VENTURE PHOTON 100 CMOS diffractometer	4560 independent reflections
Radiation source: INCOATEC IµS micro–focus source	3185 reflections with *I* > 2σ(*I*)
Mirror monochromator	*R*_int_ = 0.105
Detector resolution: 10.4167 pixels mm^-1^	θ_max_ = 68.4°, θ_min_ = 2.9°
ω scans	*h* = −23→24
Absorption correction: multi-scan (*SADABS*; Bruker, 2012)	*k* = −7→7
*T*_min_ = 0.84, *T*_max_ = 0.96	*l* = −24→24
32772 measured reflections	

### Refinement

**Table:**

Refinement on *F*^2^	Primary atom site location: structure-invariant direct methods
Least-squares matrix: full	Secondary atom site location: difference Fourier map
*R*[*F*^2^ > 2σ(*F*^2^)] = 0.054	Hydrogen site location: mixed
*wR*(*F*^2^) = 0.111	H-atom parameters constrained
*S* = 1.08	*w* = 1/[σ^2^(*F*_o_^2^) + (0.021*P*)^2^ + 2.7033*P*] where *P* = (*F*_o_^2^ + 2*F*_c_^2^)/3
4560 reflections	(Δ/σ)_max_ < 0.001
361 parameters	Δρ_max_ = 0.22 e Å^−^^3^
0 restraints	Δρ_min_ = −0.24 e Å^−^^3^

### Special details

**Table:**

Geometry. All e.s.d.'s (except the e.s.d. in the dihedral angle between two l.s. planes) are estimated using the full covariance matrix. The cell e.s.d.'s are taken into account individually in the estimation of e.s.d.'s in distances, angles and torsion angles; correlations between e.s.d.'s in cell parameters are only used when they are defined by crystal symmetry. An approximate (isotropic) treatment of cell e.s.d.'s is used for estimating e.s.d.'s involving l.s. planes.
Refinement. Refinement of *F*^2^ against ALL reflections. The weighted *R*-factor *wR* and goodness of fit *S* are based on *F*^2^, conventional *R*-factors *R* are based on *F*, with *F* set to zero for negative *F*^2^. The threshold expression of *F*^2^ > σ(*F*^2^) is used only for calculating *R*-factors(gt) *etc*. and is not relevant to the choice of reflections for refinement. *R*-factors based on *F*^2^ are statistically about twice as large as those based on *F*, and *R*- factors based on ALL data will be even larger. H-atoms attached to carbon were placed in calculated positions (C—H = 0.95 Å) while those attached to nitrogen were placed in locations derived from a difference map and their coordinates adjusted to give N—H = 0.91 Å. All were included as riding contributions with isotropic displacement parameters 1.5 times those of the attached atoms.

### Fractional atomic coordinates and isotropic or equivalent isotropic displacement parameters (Å^2^)

**Table:**

	*x*	*y*	*z*	*U*_iso_*/*U*_eq_	
O1	0.78438 (9)	0.0796 (3)	0.48058 (9)	0.0231 (4)	
O2	0.90255 (9)	0.6421 (3)	0.40602 (9)	0.0254 (4)	
N1	0.80979 (10)	0.6347 (3)	0.46106 (10)	0.0201 (5)	
H1	0.8013	0.7785	0.4655	0.024*	
N2	0.85417 (10)	0.3212 (3)	0.43843 (10)	0.0198 (5)	
N3	0.90346 (11)	0.1794 (4)	0.42044 (11)	0.0255 (5)	
H3A	0.9247	0.1205	0.4586	0.031*	
H3B	0.8834	0.0767	0.3923	0.031*	
C1	0.77157 (13)	0.4751 (4)	0.49361 (12)	0.0189 (6)	
C2	0.80275 (13)	0.2652 (4)	0.47094 (12)	0.0194 (6)	
C3	0.86045 (13)	0.5491 (4)	0.43294 (12)	0.0205 (6)	
C4	0.69599 (13)	0.4923 (4)	0.47123 (12)	0.0191 (6)	
C5	0.65335 (13)	0.3213 (5)	0.48164 (13)	0.0255 (6)	
H5	0.6714	0.1904	0.5012	0.031*	
C6	0.58517 (14)	0.3413 (5)	0.46371 (14)	0.0303 (7)	
H6C	0.5566	0.2234	0.4708	0.036*	
C7	0.55780 (15)	0.5314 (5)	0.43552 (14)	0.0316 (7)	
H7	0.5108	0.5442	0.4231	0.038*	
C8	0.59973 (14)	0.7018 (5)	0.42579 (14)	0.0299 (7)	
H8	0.5814	0.8336	0.4071	0.036*	
C9	0.66835 (14)	0.6820 (4)	0.44302 (13)	0.0246 (6)	
H9	0.6968	0.7998	0.4354	0.030*	
C10	0.78600 (12)	0.4917 (4)	0.56998 (13)	0.0201 (6)	
C11	0.80444 (13)	0.6912 (4)	0.59968 (13)	0.0255 (6)	
H11	0.8090	0.8148	0.5727	0.031*	
C12	0.81613 (14)	0.7099 (5)	0.66814 (14)	0.0309 (7)	
H12	0.8284	0.8466	0.6877	0.037*	
C13	0.81028 (14)	0.5327 (5)	0.70839 (14)	0.0294 (7)	
H13	0.8190	0.5462	0.7553	0.035*	
C14	0.79153 (14)	0.3359 (5)	0.67955 (14)	0.0291 (7)	
H14	0.7872	0.2131	0.7069	0.035*	
C15	0.77886 (14)	0.3151 (5)	0.61077 (13)	0.0259 (6)	
H15	0.7652	0.1789	0.5916	0.031*	
O3	0.93849 (10)	0.0679 (3)	0.56241 (9)	0.0279 (5)	
O4	0.99521 (9)	0.6386 (3)	0.70282 (9)	0.0235 (4)	
N4	0.97628 (11)	0.0837 (3)	0.67430 (10)	0.0218 (5)	
H4	0.9832	−0.0606	0.6816	0.026*	
N5	0.96189 (10)	0.3934 (3)	0.62039 (10)	0.0195 (5)	
N6	0.95376 (11)	0.5316 (3)	0.56462 (10)	0.0240 (5)	
H6A	0.9316	0.6529	0.5750	0.029*	
H6B	0.9960	0.5739	0.5595	0.029*	
C16	0.99947 (13)	0.2460 (4)	0.72474 (12)	0.0200 (6)	
C17	0.98609 (12)	0.4523 (4)	0.68303 (12)	0.0181 (6)	
C18	0.95735 (13)	0.1661 (4)	0.61355 (13)	0.0206 (6)	
C19	1.07440 (13)	0.2216 (4)	0.75053 (12)	0.0195 (6)	
C20	1.10590 (14)	0.0225 (4)	0.74733 (14)	0.0264 (6)	
H20	1.0815	−0.0973	0.7270	0.032*	
C21	1.17274 (14)	−0.0036 (5)	0.77354 (15)	0.0303 (7)	
H21	1.1935	−0.1411	0.7710	0.036*	
C22	1.20901 (14)	0.1669 (5)	0.80305 (13)	0.0283 (7)	
H22	1.2547	0.1484	0.8209	0.034*	
C23	1.17813 (14)	0.3665 (5)	0.80648 (14)	0.0307 (7)	
H23	1.2029	0.4858	0.8266	0.037*	
C24	1.11154 (14)	0.3936 (4)	0.78078 (13)	0.0264 (6)	
H24	1.0909	0.5311	0.7838	0.032*	
C25	0.95806 (13)	0.2439 (4)	0.78283 (12)	0.0216 (6)	
C26	0.95771 (14)	0.4223 (5)	0.82471 (13)	0.0264 (6)	
H26	0.9832	0.5473	0.8173	0.032*	
C27	0.92022 (14)	0.4189 (5)	0.87740 (14)	0.0322 (7)	
H27	0.9204	0.5415	0.9057	0.039*	
C28	0.88276 (14)	0.2383 (5)	0.88873 (14)	0.0355 (8)	
H28	0.8567	0.2373	0.9243	0.043*	
C29	0.88340 (15)	0.0586 (5)	0.84782 (15)	0.0347 (7)	
H29	0.8579	−0.0663	0.8555	0.042*	
C30	0.92130 (13)	0.0610 (5)	0.79549 (13)	0.0267 (6)	
H30	0.9221	−0.0635	0.7681	0.032*	

### Atomic displacement parameters (Å^2^)

**Table:**

	*U*^11^	*U*^22^	*U*^33^	*U*^12^	*U*^13^	*U*^23^
O1	0.0258 (10)	0.0146 (10)	0.0292 (10)	−0.0005 (8)	0.0052 (8)	−0.0005 (8)
O2	0.0259 (10)	0.0235 (10)	0.0284 (10)	−0.0002 (8)	0.0102 (9)	0.0043 (8)
N1	0.0208 (12)	0.0124 (11)	0.0292 (12)	0.0016 (9)	0.0107 (10)	0.0000 (9)
N2	0.0195 (12)	0.0135 (11)	0.0270 (12)	0.0036 (9)	0.0055 (10)	−0.0002 (9)
N3	0.0281 (13)	0.0198 (12)	0.0298 (13)	0.0066 (10)	0.0081 (11)	−0.0010 (10)
C1	0.0213 (14)	0.0136 (13)	0.0226 (13)	−0.0012 (11)	0.0060 (11)	−0.0004 (10)
C2	0.0205 (14)	0.0168 (14)	0.0201 (13)	0.0014 (11)	−0.0007 (11)	−0.0001 (11)
C3	0.0241 (15)	0.0180 (14)	0.0198 (13)	0.0009 (12)	0.0045 (12)	0.0005 (11)
C4	0.0206 (14)	0.0190 (14)	0.0183 (13)	0.0000 (11)	0.0052 (11)	−0.0046 (11)
C5	0.0236 (15)	0.0216 (15)	0.0328 (16)	−0.0013 (12)	0.0088 (13)	−0.0022 (12)
C6	0.0254 (16)	0.0313 (17)	0.0346 (17)	−0.0070 (13)	0.0057 (13)	−0.0034 (13)
C7	0.0234 (16)	0.0431 (19)	0.0278 (16)	0.0010 (14)	0.0014 (13)	−0.0044 (14)
C8	0.0256 (16)	0.0341 (17)	0.0287 (15)	0.0066 (13)	−0.0006 (13)	0.0045 (13)
C9	0.0257 (15)	0.0241 (15)	0.0239 (14)	−0.0011 (12)	0.0026 (12)	0.0022 (11)
C10	0.0153 (13)	0.0207 (14)	0.0247 (14)	0.0014 (11)	0.0040 (11)	−0.0021 (11)
C11	0.0267 (15)	0.0204 (15)	0.0281 (15)	0.0003 (12)	−0.0006 (13)	0.0005 (12)
C12	0.0323 (17)	0.0249 (16)	0.0331 (16)	0.0027 (13)	−0.0041 (14)	−0.0077 (13)
C13	0.0242 (16)	0.0375 (18)	0.0254 (15)	0.0082 (13)	−0.0001 (12)	−0.0067 (13)
C14	0.0291 (16)	0.0319 (17)	0.0282 (15)	0.0054 (13)	0.0101 (13)	0.0050 (13)
C15	0.0283 (16)	0.0215 (15)	0.0292 (15)	0.0014 (12)	0.0088 (13)	0.0004 (12)
O3	0.0360 (12)	0.0214 (10)	0.0247 (10)	−0.0002 (9)	−0.0018 (9)	−0.0042 (8)
O4	0.0294 (11)	0.0136 (10)	0.0275 (10)	−0.0019 (8)	0.0035 (8)	−0.0010 (8)
N4	0.0302 (13)	0.0122 (11)	0.0214 (12)	−0.0008 (10)	−0.0024 (10)	0.0014 (9)
N5	0.0217 (12)	0.0153 (11)	0.0219 (11)	0.0006 (9)	0.0040 (10)	0.0016 (9)
N6	0.0259 (13)	0.0206 (12)	0.0254 (12)	0.0031 (10)	0.0040 (10)	0.0070 (10)
C16	0.0246 (15)	0.0122 (13)	0.0226 (13)	−0.0020 (11)	0.0017 (11)	−0.0024 (11)
C17	0.0150 (13)	0.0187 (14)	0.0208 (13)	0.0000 (11)	0.0036 (11)	−0.0008 (11)
C18	0.0194 (14)	0.0177 (14)	0.0244 (14)	−0.0005 (11)	0.0014 (12)	−0.0004 (11)
C19	0.0224 (14)	0.0196 (14)	0.0171 (13)	0.0010 (11)	0.0045 (11)	0.0010 (11)
C20	0.0256 (16)	0.0180 (15)	0.0361 (16)	−0.0013 (12)	0.0060 (13)	−0.0017 (12)
C21	0.0273 (16)	0.0201 (15)	0.0444 (18)	0.0040 (12)	0.0074 (14)	0.0044 (13)
C22	0.0234 (15)	0.0331 (17)	0.0277 (15)	0.0026 (13)	0.0009 (13)	0.0051 (12)
C23	0.0282 (16)	0.0295 (17)	0.0331 (16)	−0.0020 (13)	−0.0006 (13)	−0.0063 (13)
C24	0.0259 (16)	0.0214 (15)	0.0306 (15)	0.0015 (12)	−0.0010 (13)	−0.0049 (12)
C25	0.0196 (14)	0.0218 (14)	0.0223 (13)	0.0008 (11)	−0.0005 (11)	0.0048 (11)
C26	0.0273 (16)	0.0259 (15)	0.0259 (15)	0.0023 (13)	0.0025 (12)	0.0013 (12)
C27	0.0294 (17)	0.0413 (19)	0.0266 (15)	0.0058 (14)	0.0064 (13)	0.0005 (13)
C28	0.0249 (16)	0.055 (2)	0.0269 (16)	0.0029 (15)	0.0056 (13)	0.0110 (15)
C29	0.0253 (16)	0.0420 (19)	0.0357 (17)	−0.0092 (14)	−0.0004 (14)	0.0117 (15)
C30	0.0225 (15)	0.0284 (16)	0.0284 (15)	−0.0046 (12)	0.0001 (12)	0.0059 (12)

### Geometric parameters (Å, º)

**Table:**

O1—C2	1.226 (3)	O3—C18	1.218 (3)
O2—C3	1.214 (3)	O4—C17	1.222 (3)
N1—C3	1.344 (3)	N4—C18	1.342 (3)
N1—C1	1.462 (3)	N4—C16	1.463 (3)
N1—H1	0.9100	N4—H4	0.9100
N2—C2	1.347 (3)	N5—C17	1.350 (3)
N2—N3	1.408 (3)	N5—N6	1.410 (3)
N2—C3	1.417 (3)	N5—C18	1.410 (3)
N3—H3A	0.9100	N6—H6A	0.9101
N3—H3B	0.9100	N6—H6B	0.9099
C1—C4	1.533 (4)	C16—C17	1.532 (3)
C1—C2	1.536 (3)	C16—C25	1.536 (3)
C1—C10	1.543 (3)	C16—C19	1.537 (4)
C4—C9	1.386 (4)	C19—C20	1.388 (4)
C4—C5	1.394 (4)	C19—C24	1.392 (4)
C5—C6	1.379 (4)	C20—C21	1.389 (4)
C5—H5	0.9500	C20—H20	0.9500
C6—C7	1.386 (4)	C21—C22	1.371 (4)
C6—H6C	0.9500	C21—H21	0.9500
C7—C8	1.379 (4)	C22—C23	1.385 (4)
C7—H7	0.9500	C22—H22	0.9500
C8—C9	1.385 (4)	C23—C24	1.383 (4)
C8—H8	0.9500	C23—H23	0.9500
C9—H9	0.9500	C24—H24	0.9500
C10—C15	1.388 (4)	C25—C26	1.392 (4)
C10—C11	1.398 (4)	C25—C30	1.392 (4)
C11—C12	1.384 (4)	C26—C27	1.392 (4)
C11—H11	0.9500	C26—H26	0.9500
C12—C13	1.379 (4)	C27—C28	1.382 (4)
C12—H12	0.9500	C27—H27	0.9500
C13—C14	1.378 (4)	C28—C29	1.386 (4)
C13—H13	0.9500	C28—H28	0.9500
C14—C15	1.392 (4)	C29—C30	1.391 (4)
C14—H14	0.9500	C29—H29	0.9500
C15—H15	0.9500	C30—H30	0.9500
			
C3—N1—C1	113.9 (2)	C18—N4—C16	114.2 (2)
C3—N1—H1	126.1	C18—N4—H4	122.6
C1—N1—H1	119.5	C16—N4—H4	121.8
C2—N2—N3	125.8 (2)	C17—N5—N6	125.7 (2)
C2—N2—C3	112.1 (2)	C17—N5—C18	111.8 (2)
N3—N2—C3	121.4 (2)	N6—N5—C18	121.5 (2)
N2—N3—H3A	107.0	N5—N6—H6A	108.6
N2—N3—H3B	109.0	N5—N6—H6B	104.8
H3A—N3—H3B	112.1	H6A—N6—H6B	106.7
N1—C1—C4	112.5 (2)	N4—C16—C17	99.47 (19)
N1—C1—C2	99.74 (19)	N4—C16—C25	112.2 (2)
C4—C1—C2	113.6 (2)	C17—C16—C25	111.1 (2)
N1—C1—C10	111.8 (2)	N4—C16—C19	112.2 (2)
C4—C1—C10	109.8 (2)	C17—C16—C19	111.2 (2)
C2—C1—C10	109.2 (2)	C25—C16—C19	110.4 (2)
O1—C2—N2	125.9 (2)	O4—C17—N5	125.6 (2)
O1—C2—C1	126.4 (2)	O4—C17—C16	126.2 (2)
N2—C2—C1	107.7 (2)	N5—C17—C16	108.2 (2)
O2—C3—N1	128.6 (2)	O3—C18—N4	127.9 (2)
O2—C3—N2	125.4 (2)	O3—C18—N5	125.9 (2)
N1—C3—N2	105.9 (2)	N4—C18—N5	106.2 (2)
C9—C4—C5	118.6 (3)	C20—C19—C24	118.2 (2)
C9—C4—C1	120.6 (2)	C20—C19—C16	120.3 (2)
C5—C4—C1	120.7 (2)	C24—C19—C16	121.4 (2)
C6—C5—C4	120.3 (3)	C19—C20—C21	120.7 (3)
C6—C5—H5	119.8	C19—C20—H20	119.6
C4—C5—H5	119.8	C21—C20—H20	119.6
C5—C6—C7	120.8 (3)	C22—C21—C20	120.7 (3)
C5—C6—H6C	119.6	C22—C21—H21	119.6
C7—C6—H6C	119.6	C20—C21—H21	119.6
C8—C7—C6	119.1 (3)	C21—C22—C23	119.1 (3)
C8—C7—H7	120.5	C21—C22—H22	120.4
C6—C7—H7	120.5	C23—C22—H22	120.4
C7—C8—C9	120.5 (3)	C24—C23—C22	120.5 (3)
C7—C8—H8	119.8	C24—C23—H23	119.7
C9—C8—H8	119.8	C22—C23—H23	119.7
C8—C9—C4	120.7 (3)	C23—C24—C19	120.8 (3)
C8—C9—H9	119.7	C23—C24—H24	119.6
C4—C9—H9	119.7	C19—C24—H24	119.6
C15—C10—C11	118.4 (2)	C26—C25—C30	118.7 (3)
C15—C10—C1	121.9 (2)	C26—C25—C16	120.8 (2)
C11—C10—C1	119.7 (2)	C30—C25—C16	120.5 (2)
C12—C11—C10	120.4 (3)	C25—C26—C27	120.5 (3)
C12—C11—H11	119.8	C25—C26—H26	119.8
C10—C11—H11	119.8	C27—C26—H26	119.8
C13—C12—C11	120.9 (3)	C28—C27—C26	120.4 (3)
C13—C12—H12	119.5	C28—C27—H27	119.8
C11—C12—H12	119.5	C26—C27—H27	119.8
C14—C13—C12	119.0 (3)	C27—C28—C29	119.6 (3)
C14—C13—H13	120.5	C27—C28—H28	120.2
C12—C13—H13	120.5	C29—C28—H28	120.2
C13—C14—C15	120.7 (3)	C28—C29—C30	120.0 (3)
C13—C14—H14	119.6	C28—C29—H29	120.0
C15—C14—H14	119.6	C30—C29—H29	120.0
C10—C15—C14	120.5 (3)	C29—C30—C25	120.7 (3)
C10—C15—H15	119.7	C29—C30—H30	119.6
C14—C15—H15	119.7	C25—C30—H30	119.6
			
C3—N1—C1—C4	−128.7 (2)	C18—N4—C16—C17	3.9 (3)
C3—N1—C1—C2	−8.0 (3)	C18—N4—C16—C25	121.4 (2)
C3—N1—C1—C10	107.3 (2)	C18—N4—C16—C19	−113.7 (2)
N3—N2—C2—O1	−12.5 (4)	N6—N5—C17—O4	11.6 (4)
C3—N2—C2—O1	177.1 (2)	C18—N5—C17—O4	180.0 (2)
N3—N2—C2—C1	167.5 (2)	N6—N5—C17—C16	−168.9 (2)
C3—N2—C2—C1	−2.9 (3)	C18—N5—C17—C16	−0.5 (3)
N1—C1—C2—O1	−173.8 (2)	N4—C16—C17—O4	177.7 (3)
C4—C1—C2—O1	−53.9 (3)	C25—C16—C17—O4	59.4 (3)
C10—C1—C2—O1	68.9 (3)	C19—C16—C17—O4	−63.9 (3)
N1—C1—C2—N2	6.2 (2)	N4—C16—C17—N5	−1.9 (3)
C4—C1—C2—N2	126.1 (2)	C25—C16—C17—N5	−120.2 (2)
C10—C1—C2—N2	−111.1 (2)	C19—C16—C17—N5	116.5 (2)
C1—N1—C3—O2	−175.5 (3)	C16—N4—C18—O3	177.1 (3)
C1—N1—C3—N2	6.7 (3)	C16—N4—C18—N5	−4.4 (3)
C2—N2—C3—O2	180.0 (3)	C17—N5—C18—O3	−178.5 (3)
N3—N2—C3—O2	9.1 (4)	N6—N5—C18—O3	−9.5 (4)
C2—N2—C3—N1	−2.1 (3)	C17—N5—C18—N4	2.9 (3)
N3—N2—C3—N1	−173.0 (2)	N6—N5—C18—N4	171.9 (2)
N1—C1—C4—C9	−21.0 (3)	N4—C16—C19—C20	−25.0 (3)
C2—C1—C4—C9	−133.3 (2)	C17—C16—C19—C20	−135.4 (2)
C10—C1—C4—C9	104.2 (3)	C25—C16—C19—C20	100.9 (3)
N1—C1—C4—C5	162.1 (2)	N4—C16—C19—C24	158.5 (2)
C2—C1—C4—C5	49.7 (3)	C17—C16—C19—C24	48.1 (3)
C10—C1—C4—C5	−72.8 (3)	C25—C16—C19—C24	−75.6 (3)
C9—C4—C5—C6	0.4 (4)	C24—C19—C20—C21	−0.1 (4)
C1—C4—C5—C6	177.5 (2)	C16—C19—C20—C21	−176.8 (2)
C4—C5—C6—C7	−0.4 (4)	C19—C20—C21—C22	−0.1 (4)
C5—C6—C7—C8	−0.3 (4)	C20—C21—C22—C23	0.0 (4)
C6—C7—C8—C9	1.0 (4)	C21—C22—C23—C24	0.3 (4)
C7—C8—C9—C4	−1.0 (4)	C22—C23—C24—C19	−0.5 (4)
C5—C4—C9—C8	0.3 (4)	C20—C19—C24—C23	0.4 (4)
C1—C4—C9—C8	−176.8 (2)	C16—C19—C24—C23	177.0 (2)
N1—C1—C10—C15	−153.5 (2)	N4—C16—C25—C26	−160.2 (2)
C4—C1—C10—C15	80.9 (3)	C17—C16—C25—C26	−49.8 (3)
C2—C1—C10—C15	−44.2 (3)	C19—C16—C25—C26	73.9 (3)
N1—C1—C10—C11	28.9 (3)	N4—C16—C25—C30	21.3 (3)
C4—C1—C10—C11	−96.7 (3)	C17—C16—C25—C30	131.6 (3)
C2—C1—C10—C11	138.3 (2)	C19—C16—C25—C30	−104.6 (3)
C15—C10—C11—C12	0.9 (4)	C30—C25—C26—C27	−1.2 (4)
C1—C10—C11—C12	178.6 (2)	C16—C25—C26—C27	−179.8 (2)
C10—C11—C12—C13	0.4 (4)	C25—C26—C27—C28	−0.1 (4)
C11—C12—C13—C14	−1.0 (4)	C26—C27—C28—C29	0.9 (4)
C12—C13—C14—C15	0.2 (4)	C27—C28—C29—C30	−0.4 (4)
C11—C10—C15—C14	−1.6 (4)	C28—C29—C30—C25	−1.0 (4)
C1—C10—C15—C14	−179.3 (2)	C26—C25—C30—C29	1.8 (4)
C13—C14—C15—C10	1.1 (4)	C16—C25—C30—C29	−179.6 (2)

### Hydrogen-bond geometry (Å, º)

**Table:**

*D*—H···*A*	*D*—H	H···*A*	*D*···*A*	*D*—H···*A*
N1—H1···O1^i^	0.91	1.92	2.828 (3)	177
N3—H3*A*···O3	0.91	2.11	2.957 (3)	154
N4—H4···O4^ii^	0.91	1.91	2.820 (3)	176
N6—H6*A*···O3^i^	0.91	2.58	3.320 (3)	139
N6—H6*B*···O2^iii^	0.91	2.46	3.070 (3)	124
N6—H6*B*···N3^iii^	0.91	2.52	3.363 (3)	154

Symmetry codes: (i) *x*, *y*+1, *z*; (ii) *x*, *y*−1, *z*; (iii) −*x*+2, −*y*+1, −*z*+1.
